# Direct molecular detection of a broad range of bacterial and viral organisms and *Streptococcus pneumoniae* vaccine serotypes in children with otitis media with effusion

**DOI:** 10.1186/s13104-016-2040-4

**Published:** 2016-04-29

**Authors:** Robert Slinger, Melanie Duval, Jonathan Langill, Matthew Bromwich, Johnna MacCormick, Francis Chan, Jean-Philippe Vaccani

**Affiliations:** Department of Laboratory Medicine and Pathology, Children’s Hospital of Eastern Ontario, University of Ottawa, 401 Smyth Rd, Ottawa, ON K1H 8L1 Canada; Department of Surgery, Children’s Hospital of Eastern Ontario, University of Ottawa, Ottawa, ON Canada

**Keywords:** Otitis media with effusion, PCR, Bacteria, Viruses, *Streptococcus pneumoniae*

## Abstract

**Background:**

Otitis media with effusion (OME) causes significant morbidity in children, but the causes of OME and methods for prevention are unclear. To look for potential infectious etiologies, we performed a pilot study using multiple-target real-time polymerase chain reaction (qPCR) for 27 infectious agents, including nine bacterial organisms and 18 respiratory viruses in middle ear fluids (MEFs) from children with OME. QPCR was also performed for the 13 *Streptococcus pneumoniae* serotypes contained in the current vaccine.

**Results:**

Forty-eight MEF samples were obtained and qPCR detected bacterial nucleic acid (NA) in 39/48 (81 %) and viral NA in 7/48 (15 %). *Alloiococcus otitidis* and *S. pneumoniae* were both detected in 15/48 (31 %) MEFs, followed by *M. catarrhalis* in 14/48 (29 %), *H. influenzae* in 5/48 (10 %) and *M. pneumoniae* in 4/48 (8 %). Rhinoviruses were most common virus type detected, found in 4/48 (8 %) MEFs. Serotypes included in the current 13-serotype vaccine were detected in only 3/15 (20 %) *S. pneumoniae* qPCR-positive MEFs.

**Conclusions:**

Bacteria may play an important role in OME, since over 80 % of MEFs contained bacterial NA. Further research into the role of *A. otitidis* in OME will be helpful. Serotypes of *S. pneumoniae* not included in the current 13-serotype vaccine may be involved in OME. Larger studies of OME *S. pneumoniae* serotypes are needed to help determine which additional serotypes should be included in future vaccine formulations in order to try to prevent OME.

## Background

Otitis media with effusion (OME) is a common childhood condition in which fluid persists in the middle ear cavity for 3 months or more. It has been estimated that up to 80 % of children have been affected by OME at some time by the age of four [1]. Many children suffering from this condition will have a mild to moderate hearing loss, averaging 27 decibels, which is sufficient to lead to language development delay [2].

While the exact pathogenesis of OME remains unknown, multiple hypotheses have been put forward. The position and length of the Eustachian tube in children and a persistent inflammatory process in the middle ear cavity due to frequent upper respiratory tract infections are both thought to contribute to the frequency of OME in childhood [1].

Since bacterial cultures of middle ear fluid (MEF) are negative in the majority of children with OME, OME was once thought to be an inflammatory but non-infectious condition. However, with the use of more sensitive molecular detection methods such as polymerase-chain reaction (PCR), studies have shown that many bacterial culture-negative OME specimens contain bacterial DNA [3–6].

Based on studies by Hall-Stoodley and others [7], it is now thought that bacteria persist chronically in the middle ear in the form of biofilms in many or most children with OME. Biofilms are known to be difficult to eradicate with antimicrobial therapy, which could account for the persistence of effusions in children treated with antibiotics for OME in clinical trials [8]. The biofilm hypothesis would also fit with the observation that bacterial cultures of MEF are often negative, as bacteria in biofilms are more difficult to culture than free-floating (planktonic) bacteria [8, 9].

Bacteria detected by molecular methods from OME middle ear fluid include those known to cause acute otitis media (AOM), namely, *Streptococcus pneumoniae,Haemophilus influenzae* and *Moraxella catarrhalis* [3–6]. As well as accepted otitis pathogens, other bacteria whose pathogenicity is the middle ear is uncertain, such as *Alloiococcus otitidis* and *Helicobacter pylori* have also been detected by molecular studies in OME samples [10–13].

Viruses have also been detected in some MEF from OME. Viral RNA was detected in approximately 1/3 of MEF samples from children undergoing tympanostomy tube insertion [14, 15]. Viruses detected by PCR have included rhinoviruses, enteroviruses, respiratory syncytial virus (RSV), and human coronaviruses [14–16].

In order to determine the prevalence of bacterial and viral agents in children with OME in our region, we performed a pilot study using real-time PCR (qPCR) for twenty-seven bacterial and viral pathogens in MEF samples. Also, for the first time to our knowledge, MEFs were also tested directly for the 13 serotypes contained in the conjugate pneumococcal vaccine (Prevnar 13, Pfizer) to assess the potential impact of the 13-serotype *S. pneumoniae* on OME associated with this bacterium [17]. Our overall objectives were to better understand the microbiology of OME in children, and to determine if the current conjugated pneumococcal vaccine would likely lead to prevention of OME.

## Methods

### Study outcome measures

1. Presence or absence of the bacteria and viruses listed in Table 1 as determined by real-time PCR from OME fluids.

**Table 1:** Real-time PCR assays for bacteria, viruses and *Streptococcus pneumoniae* serotypes

Target	Forward primer	Reverse primer	Probe (all with MGB except^a^)	Ref.
*S. pneumoniae*	acgcaatctagcagatga agca	tcgtgcgttttaattccagct	tgccgaaaacgcttgatacagggag^a^	[19]
*H. influenzae*	ggttaaatatgccgatggtgttg	tgcatctttacgcacggtgta	ttgtgtacactccgttggtaaaagaacttgcac^a^	[19]
*A. otitidis*	ctacgcatttcaccgctacac	ggggaagaacacggatagga	agtccgccagtttccaatgccgttcca^a^	[10]^b^
*H. pylori*	cgtggcaagcatgatccat	gggtatgcacggttacgagttt	tcaggaaacatcgcttcaatacccactt^a^	[27]
*M. catarrhalis*	gtcaaacagctggaggtattgc	gacatgatgctcacctgctcta	atcgcaattgcaacttt	[28]
*C. pneumoniae*	cgcgaaggaccttacctgga	gtatctgtccttgcggaaagct	ctacagttgtcaaatacatgtc	[20]
*M. pneumoniae*	cgtggtgaagtgaaacatctcagtag	gcaagccctacaacccctatcta	atgataagtttggcctgttc	[20]
*B. pertussis*	catcaagcaccgctttaccc	tgttgggagttctggtaggtgtg	cttaccgcccacagac	[20]
*B. parapertussis*	gatatcaacgggtgacggatc	gcctgcacgttgctcgat	cagcaattggctgcagg	[20]
SP serotype 1	cgtgcggtaattgaagctatga	tgtggccccagcaactct	cttgcccttgtatagggt	[21]
SP serotype 3	ggtcagcagaaagtatgcattgg	tcgtttatccagggtctgatga	tattggatgtggtttatcgtgaag	[21]
SP serotype 4	cggcaggcaaaccaattat	catctcgttcgggactaaca	caggagatgctaaaata	[21]
SP serotype 5	ttacgggagtatcttatgtctttaatgg	cagcattccagtagcctaaaactaga	tctcagcaactctatttgg	[21]
SP serotype 6A	gctagagatggttccttcagttgat	catactctagtgcaaactttgcaaaat	ctggctcatgatagtt	[21]
SP serotype 6B	gctagagatggttccttcagttgat	catactctagtgcaaactttgcaaaat	actgtctcatgataatt	[21]
SP serotype 7F/A	aagcacagtgcgtgaacaat	aaaatctccctgtcccttcc	ctattccagaagaatctc	[21]
SP serotype 9 V/A	tggaatgggcaaagggtagta	tcggttccccaagattttctc	ttaatcatgctaacggctcat	[21]
SP serotype 14	cgactgaaatgtcactaggagaagat	aatacagtccatcaattactgcaatactc	attcgtttgccaatacttga	[21]
SP serotype 18C/B	ccctgaaactagttgggaaca	ttccaatcatcacccattaca	aaagtcagatgttaaagactac	[21]
SP serotype 19A	gctgtgtttatgggggttgg	agagacgtttaggctcatttgc	atgcaaaatgctcacctag	[21]
SP serotype 19F/B/C	aattcggtatttatgggagttgg	agagacgtttaggctcattagc	atgcaaaagtcaaatttaga	[21]
SP serotype 23F	ctgggccaagatatttaaaagagagt	aattccgcatcagagtatgcaa	ttgctcttcgaaaaatgt	[21]
RSV A	ctcaatttcctcacttctccagtgt	cttgattcctcggtgtacctctgt	cattatgcctaggccagcag	[20]
RSV B	ttcctaacttctcaagtgtggtccta	ctggtttcttggcgtacctctatac	tcccattatgcctagacct	[20]
Influenza A	ccccctcaaagccgagat	caagattggtcttgtctttagcca	ccatgagagcctcaagat	[20]
Influenza B	aatacggtggattaaacaaaagcaa	caggaggtctatatttggttccatt	catattgggcaatttccta	[20
HMPV A	cacctgagtgatttatcatacaagca	ttagcatacagaatttctccacacaa	acaccctcatcattgc	[20] MGB
HMPV B	caagaacaaatgtgacattgctgat	gaaaactgccgcacaacatttag	aagctgacagccatct	[20]
Rhinovirus	caagtaatggacagggtgtgaagag	ccaaagtagtcggtcccatcc	tccggcccctgaat	[20]
Enterovirus	gcccctgaatgcggctaa	ggaaacacggacacccaaagta	tctgcagcggaacc	[20]
Bocavirus	ggcagaattcagccatactcaaa	tctgggttagtgcaaaccatga	ccacagtcatcagacact	[20] MGB
Adenovirus	ccacggtggggtttctaaactt	cccagtggtcttacatgcacatc	tgcaccagacccgg	[20]
Coronavirus OC43	cgatgaggctattccgactaggt	ccttcctgagccttcaatatagtaacc	tccgcctggcacggt	[20]
Coronavirus 229E	ttccgacgtgctcgaacttt	ccaacacggttgtgacagtga	tcctgaggtcaatgca	[20]
Coronavirus HKU1	gccttgcgaatgaatgtgct	tgcatcaccactgctagtaccac	ttgctattatgttaagcctg	[20] MGB
Coronavirus NL63	ggaagcgtgttcctaccagaga	agcaagctgtggaaaacctttg	caaagcactgaataac	[20]
PIV1	acagatgaaattttcaagtgctactttagt	gcctcttttaatgccatattatcattaga	atggtaataaatcgactcgct	[20]
PIV 2	tgcatgttttataactactgatcttgctaa	gttcgagcaaaatggattatggt	actgtcttcaatggagatat	[20]
PIV 3	tgctgttcgatgccaacaa	attttatgctcctatctagtggaagaca	ttgctcttgctcctca	[20]
PIV 4	cctggagtcccatccaaagtaag	tgagactgttattttaagtgcatctatacg	tttgttgatcaagacaatac	[20]

*RSV* Respiratory syncytial virus; *PIV* Parainfluenza virus; *HMPV* human metapneumovirus; *SP* *S. pneumoniae*

^a^ Probes contained an internal quencher and no minor groove binder. All other probes contained a minor groove binder and no internal quencher

^b^ Primers from published reference, probe this study
2. Comparison of PCR and culture results for bacterial detection from OME fluids.
3. For *S. pneumoniae*, qPCR-positive OME fluids, the results of PCR serotyping for the serotypes contained in the 13-serotype *S. pneumoniae* vaccine.

### Study design

This was a prospective observational study performed from Oct. 2011 to Oct 2012 at the Children’s Hospital of Eastern Ontario, Ottawa, Ontario, Canada, a 165 bed tertiary care hospital serving a catchment area 1.5 million. In the province of Ontario, a 7- serotype *S. pneumoniae* vaccine was introduced for in 2002 and a 13-serotype vaccine in Dec. 2010. Both were part of the routine publicly funded immunization schedule for infants beginning at 2 months of age. Children also received infant immunization against *H. influenzae* type B.

### Ethics and subject recruitment

Ethics approval was obtained for the study from the hospital Research Ethics Board. Parents of children seen at the pediatric otolaryngology clinic at the CHEO for treatment of OME who were to undergo tympanostomy tube insertion were identified and invited to participate in the study by the treating otolaryngologist. Informed consent was sought from parents or guardians who wished to participate to allow the MEF to be collected and sent for bacterial culture and PCR studies. Immunization status, age and sex of the child were recorded from hospital records in a study form.

The inclusion criteria were patients under the age of 18 years of age requiring ventilation tube insertion for OME for whom informed consent was obtained. Patients not meeting these age, diagnosis, and consent criteria were excluded. OME was defined as the presence of fluid in the middle ear without signs or symptoms of acute ear infection that was associated with a conductive hearing loss of at least 30 decibels in two consecutive frequencies.

### Collection of MEFs

In patients who consented to participate in the study, the external auditory canal was cleaned by bathing the canal in 70 % isopropyl alcohol for 60 s. A myringotomy was then performed and middle ear fluid from each ear was suctioned into a Juhn Tym-Tap suction container. For each ear, the fluid obtained was then divided into two portions, with one portion sent for standard microbiology analysis (culture) and the second portion stored at −80 °C until for PCR testing was performed.

### Laboratory methods

#### Culture methods

OME fluids were processed using standard microbiological methods [18]. Aliquots were plated on sheep blood, chocolate, and MacConkey agar under aerobic conditions at 37 °C and examined daily for five days. An aliquot was inoculated into thioglycollate broth and aliquots were plated on anaerobic agar media and kept for 5 days under strict anaerobic conditions; these were also checked daily after being left initially for 48 h. Bacteria were identified used standard laboratory methods [18]. We did not perform viral or *Mycoplasma pneumoniae* cultures.

#### QPCR methods

Nucleic acids (NA) were extracted from MEF using an automated extraction device (iPrep, Life Technologies, Carlsbad, California). The qPCR primers and probes used for bacteria viruses, and *S. pneumoniae* serotype detection are shown in Table 1. All assays used 5′ exonuclease probes labeled at the 5′end with fluorescein amidite and with a quencher at the 3′ end.

For *A. otitidis*, we used previously published PCR primers for this bacterium [5, 10] but designed a 5′exonuclease probe for qPCR rather than using intercalating dye detection [10]. A commercial qPCR program (Allele ID, Premier Biosoft) was used to design the probe and specificity was checked using BLAST searches and by testing the assay against multiple reference strains of bacteria.

QPCR methods were performed as described previously [19, 20]. All probes were labeled with fluorescein amidite (FAM). Probes containing minor groove binders (MGB) were obtained from Life Technologies (Carlsbad, CA). All other probes were obtained from Integrated DNA Technologies (Coralville, IA) and contained a proprietary internal quencher (ZEN quencher, IDT). Briefly, singleplex real-time 5′exonuclease qPCR assays were prepared in 20 µL volumes in 96-well qPCR plate. Positive and negative control (no template) was performed with each qPCR plate run. QPCR plates were covered with MicroAmp^®^ Optical Adhesive Film (Life Technologies Carlsbad, CA) to prevent cross-contamination. QPCR was performed with a 96 well fast cycling block on a ViiA7 thermocyler (Life Technologies Inc.) using 40 cycles of 2-temperature thermocyling (95 °C × 3 s and 60 °C × 30 s).

Only samples which were positive with the *S. pneumoniae* qPCR assay were further tested using published serotype-specific qPCR assays for the 13 serotypes contained in the current conjugated vaccine (serotypes 1, 3, 4, 5, 6A, 6B, 7F, 9 V, 14, 18C, 19A, 19F, and 23F) [21]. QPCR serotyping assays were performed as above, with the exception that longer cycling times were used (40 cycles of 2-temperature thermocyling were used, with 95 °C × 15 s and 60 °C × 60 s).

## Results

Thirty-one children were enrolled in the study. One child was excluded as tympanostomy was performed for AOM rather than OME, leaving 30 that were evaluated. Forty-eight MEF samples were obtained from these 30 children. Sixteen children were female and 14 were male. The median age was 34 months with range 11–120 months. Twenty-seven of the 30 (90 %) children had received the original 7-serotype S*. pneumoniae* vaccine as part of routine infant immunization program, while three older children born prior to introduction of this vaccine had not. None had received the 13-serotype S*. pneumoniae* vaccine.

The number of MEF specimens in which bacterial and viral organisms NA were detected by qPCR, both alone and with other agents, is shown in Table 2. Overall, qPCR detected bacterial DNA in 39/48 (81 %) MEFs from 26/30 (87 %) children and detected viral NA in 7/48 (15 %) MEF from 5/30 (17 %) children. *A.otitidis* and *S. pneumoniae* DNA were both detected by qPCR in 15/48 (31 %) MEF, with *A.otitidis* in 11/30 (37 %) children and *S. pneumoniae* in 10/33 (33 %) children. These were followed by *M. catarrhalis* DNA in 14/48 (29 %) MEF from 10/30 (33 %) children, *H. influenzae* DNA in 5/48 MEF (10 %) from 4/30 (13 %) children, and *M. pneumoniae* DNA in 4/48 (8 %) MEF from 2/30 (7 %) children. Among the viruses tested for, rhinovirus RNA was present in 4/48 (8 %) MEF, coronavirus OC43 RNA in 2/48 (4 %) MEF, and influenza B RNA in 1/48 (2 %) MEF.

**Table 2:** Bacterial and viral nucleic acid detected by qPCR from middle ear fluid (MEF) samples from children undergoing tympanostomy tube insertion for otitis media with effusion

Organism name	Number of PCR-positive MEF (n = 48)	Number detected alone	Number detected with other bacteria and/or viruses
*S. pneumoniae*	15	5	10
*A.* *otitidis*	15	6	9
*M. catarrhalis*	14	9	5
*H. influenzae*	5	4	1
*M. pneumoniae*	4	2	2
Rhinoviruses	4	0	4
Coronavirus OC43 virus	2	0	2
Influenza B virus	1	1	0

Bacteria were grown in culture from 11/48 (23 %) MEF from 10/30 (33 %) children. *M. catarrhalis* was isolated from five MEF, *H. influenzae* from 3 MEF, and *S. pneumoniae* from 2 MEF. All culture positive samples for these three bacteria were also PCR-positive. One MEF grew *Streptococcus pyogenes* (group A streptococcus), a bacterium not included in the PCR testing. *A.* *otitidis* was not grown in culture, and no anaerobic bacteria were grown in culture.

With regard to detection of multiple organisms (detection of ≥ bacterial or viral NA in the same MEF), there were 28/48 (58 %) MEF with NA from a single organism detected, 6/48 (13 %) MEF with NA from 2 organisms detected and 6/48 (13 %) MEF with NA from 3 organisms detected, and 8/48 with no NA detected. Of note, 6/7 (86 %) viral- NA positive samples also had bacterial DNA detected by qPCR.

*Streptococcus pneumoniae* serotyping by qPCR detected serotype 19A DNA in MEF in 3/15 (20 %) *S. pneumoniae* qPCR-positive specimens. All three samples with serotype 19A DNA detected from children who had received the 7 serotype vaccine. Tests for the other 12 vaccine serotypes were negative.

## Discussion

The finding that over 80 % of MEF samples from children with OME contained bacterial DNA supports the idea that OME may be an infectious process. *A.otitidis* DNA was frequently detected but the pathogenicity of this organism in OME is uncertain. Several studies have reported detecting *A.* *otitidis* by PCR in MEF [10, 11]. One report suggests this bacterium may be part of the normal flora of the external ear, and not a middle-ear pathogen [22]. However, other studies show *A. otitidis* can stimulate an inflammatory response, suggesting it may cause disease in the middle ear. For example, *A.* *otitidis* elicited higher levels of inflammatory mediators than *S. pneumoniae* in a cell line model [23].

The fact that we did not isolate *A.otitidis* in culture is consistent with findings that this organism typically does not grow in culture using the methods standardly used in clinical microbiology laboratories. For example, in one study, *A. otitidis* was not grown from any MEFs but 35 % were PCR-positive [11]. Improved methods to culture this organism from MEFs are being sought [24].

As seen in other studies, *S. pneumoniae, M. catarrhalis*, and *H. influenzae* DNA were detected in a much higher proportion of MEF by PCR than culture [4–6]. This may be due to the greater sensitivity of PCR over culture, or could reflect that PCR detects both live and dead bacteria, while culture requires live bacteria. Similar to our findings, Hendolin reported 84 % of MEF were PCR-positive for bacteria while 32 % were culture positive [5], while Park reported 36.7 % MEF were PCR-positive for bacteria and 14 % of samples were positive by culture [6]. This common trend may reflect the greater sensitivity of PCR for detecting bacteria in biofilm-related infections [8].

Among the accepted bacterial pathogens, *S. pneumoniae* was detected more frequently by qPCR than *H. influenzae* or *M. catarrhalis.* Since *S. pneumoniae* could be cultured from only 2/15 (13 %) qPCR positive MEFs, culture-based detection of *S. pneumoniae* in OME may grossly underestimate the importance of this bacterium, and serotyping of positive culture samples may similarly provide very limited information. Molecular serotyping methods using qPCR or other techniques that are culture-independent thus may have be a better approach to determining which *S. pneumoniae* serotypes need to be included in future vaccines to help prevent OME. To our knowledge, this is the first study in which molecular serotyping for S*. pneumoniae* has been performed directly on MEFs from children with OME.

Serotype 19A was the only serotype of the 13 included in the latest *S. pneumoniae* vaccine that was detected in the MEF in this study. Serotype 19A was not included in the original 7-serotype vaccine, but is included in the 13 serotype vaccine, so this vaccine may decrease otitis caused by this serotype. However, our finding that only a minority of samples contained a serotype included in the new vaccine is concerning, as it suggests that non-vaccine serotypes are important in OME. In this pilot study, we were unable to perform qPCR serotyping for all of the approximately 90 *S. pneumoniae* serotypes, but we plan to test for a greater number of serotypes in subsequent studies to help determine which serotypes should be included in future vaccines.

The detection of *M. pneumoniae* PCR in several samples is also of interest. *M. pneumoniae* has been detected in OME before, but it appears to be infrequent cause. For example, Strogard reported that only 1/150 MEF was PCR positive for *M. pneumoniae* [25].

*Helicobacter pylori*, known as a cause of peptic ulcer disease, has also recently been reported into be present in MEFS of children with OME in several studies, In a study from Iran, 43 % MEFs and 25 % of adenoid samples from children undergoing myringotomy were positive for H. pylori [12]. Similarly, a study from Turkey reported detection of *H. pylori* in 47 % of MEFs from children with OME [13]. The absence of this organism in our samples may reflect geographic variability in *H. pylori* exposure in children.

In terms of viral agents, despite testing for a large number of respiratory viral agents, RNA from rhinoviruses, which are the major cause of the common cold, were the most frequently detected viruses in this study, and have also been detected in some other OME studies [14, 16]. Virus NA was detected in a much smaller proportion of MEF than bacteria and was often detected with bacterial DNA. This suggests that viruses may not be a common direct cause of OME. However, it is possible that viral infections of the upper respiratory tract could lead to Eustachian tube dysfunction, which could contribute to OME. Studies examining both nasopharyngeal and MEF samples for bacteria and viruses by qPCR may help clarify the role of viruses as possible co-factors in OME.

There are some limitations to this study that should be noted. First, the number of MEF studied was small, although the sample number was similar to that used in other work in this area [4, 5, 11]. We considered this to be a pilot study to guide future research so we elected to extensively study a smaller number of samples with a large number of PCR assays (27 microorganism assays and 13 serotype assays for *S. pneumoniae*) to guide us as to priorities for future OME research. Similarly, given the small number of *S. pneumoniae* qPCR-positive samples available for molecular serotyping, it is not possible to make definite conclusions regarding the distribution of serotypes. Nevertheless, the findings suggest that molecular serotyping will provide much more information than traditional culture-based serotyping, and also that serotypes other than those contained in the 13-valent vaccine will need to be tested for in future studies.

Second, as noted above, PCR-positive results can occur from live or dead bacterial or viral micro-organisms. However, for bacteria, Post and others have shown in an animal model that purified DNA and DNA from intact but non-viable bacteria do not persist in the middle ear. In contrast, DNA from live bacteria could be detected for 3 weeks post-middle ear inoculation even when antibiotic treatment caused bacterial culture to become negative [9].

Additional work that supports the concept that bacterial DNA detected by PCR represents live organisms comes from the study by Rayner and others [26]. This group used reverse transcriptase-polymerase chain reaction (RT-PCR) to determine if bacterial messenger RNA (mRNA) was present in pediatric OME samples that contained bacterial DNA but were sterile by standard cultural methods. Since bacterial mRNAs have a very short half-life of seconds to minutes, detection of bacteria-specific mRNAs suggests metabolically active organisms are present. This group found that 29/29 *H. influenzae* PCR- positive samples were also positive for *H. influenzae* mRNA by RT-PCR, which suggests that viable, metabolically active organisms were present in these samples.

With respect to the correlation between the detection of viral NA detection by PCR and the presence of live virus in patient samples, one study has shown that this appears to depend on the type of virus. The duration of viral shedding for influenza viruses was not significantly different when measured by culture and PCR (13 vs. 14 days, respectively). However, for RSV and parainfluenza viruses shedding lasted significantly longer by PCR than by culture. Unfortunately, rhinoviruses, the most common viruses found in our study, were not examined in this report [29].

Finally, although we tested for a wide range of organisms, there have been additional agents found in OME for which we did not perform PCR. For example, the anaerobic organism *Fusobacterium nucleatum* was found in 6/20 (30 %) OME samples using PCR in one study [30]. Although anaerobic bacterial cultures in our study were negative, it is possible that F*. nucleatum* or other anaerobic bacteria may have been detected had we used PCR for these organisms.

## Conclusion

Bacterial infections may play a role in OME since over 80 % of MEFS were qPCR-positive for bacterial organisms, while viruses were found in a much smaller proportion. Further studies to determine the pathogenicity of *A. otitidis* are needed. Additional *S. pneumoniae* serotypes not included in the 13-serotype vaccine may be important in OME, but this needs to be confirmed by larger studies.

## Abbreviations

OME: otitis media with effusion
MEF: middle ear fluid
AOM: acute otitis media
qPCR: real-time PCR
